# A magneto-elastic model for soft magnetic concentric tube robots

**DOI:** 10.1007/s11071-026-12856-3

**Published:** 2026-07-30

**Authors:** Peter Lloyd, Joshua Davy, Yael L. May, Jurgen E. Schneider, Pietro Valdastri

**Affiliations:** 1 Storm Lab, School of Electronic and Electrical Engineering, University of Leeds, Leeds, UK; 2 Leeds Institute of Cardiovascular and Metabolic Medicine (LICAMM), School of Medicine, University of Leeds, Leeds, UK

**Keywords:** Magnetic concentric tube robot, Continuum robotics, Magneto-elastic modeling, MRI-actuated catheter, Soft magnetic actuation, Bistability and hysteresis

## Abstract

This paper presents a numerical solution to a reduced-order analytical energy formulation of a soft, magnetically actuated concentric tube continuum robot. Unlike conventional concentric tube robots, which rely on pre-curved elastic energy storage and suffer from material stiffness constraints and snap through instability, our design uses softly magnetic rings adhered to braided sleeves to generate deformation directly from a strong, stationary background field. While high-field MRI systems provide a convenient actuation environment, the proposed design principle is broadly applicable to any application featuring a sufficiently strong background magnetic field (B ≳ 1T). We derive a complete magneto-elastic formulation that couples easy-plane magnetic torque with sleeve curvature to determine stable catheter configurations. The model captures mechanical hysteresis, bistability, and the nonlinear relationship between base rotation and tip pose. Experimental validation in the background field of a 7*T* pre-clinical MRI scanner demonstrates strong agreement with simulated deformation, with an overall RMS error of $4.4^\circ$. This framework provides a foundation for closed-loop control, systematic design optimization, and self-sensing capabilities in magnetically actuated soft continuum robots, enabling new classes of highly compliant robotic systems for constrained and sensitive environments.

**Supplementary Information:**

The online version contains supplementary material available at 10.1007/s11071-026-12856-3.

## Introduction

The concentric tube robot (CTR), or active cannula, was introduced in 2006 [1, 2] and offers high degree of freedom (DoF) deformation of a slender manipulator (aspect ratio up to 250) which can be fabricated at the millimeter scale [3]. This design has been demonstrated to be an effective shape-forming manipulator with tremendous clinical potential but is, by necessity, fabricated from a material with orders of magnitude higher stiffness than the living tissue through which it may navigate (the elastic modulus of nitinol E≈50,000MPa [4], that of arterial tissue E≈0.3MPa [5]). Furthermore, the CTR suffers from the snap-through instability; certain alignments of pre-bent tube offer lower energy equilibria and the CTR is prone to snap from its current state (a function of historical pose) to this lower energy pose [6]. Magnetic actuation has been proposed as a solution to this stiffness challenge, where magnetic deformation supplements or replaces mechanical pre-curvature in some [7] or all [8] of the tubes. This has lead to the emergence of the Magnetic Concentric Tube Robot (MCTR) [9–11]. The MCTR can be viewed as a form of continuum robot with in-situ reprogrammable magnetization [12, 13].

Elastomeric soft continuum manipulators (CMs) represent a promising and highly active research area among the soft robotics community [14]. A major subset of these robots are magnetically actuated CMs [15, 16] which offer the potential to usurp, at least in some part, the role of the surgical catheter. Magnetic CM’s are popular due to their extrinsic actuation, similar to the CTR, offering strong potential for miniaturization [17]. Advances in magnetic CMs have highlighted the importance of accurate, model-based descriptions. Real-time simulation enabled control relies on efficient forward models [18, 19]. At a more fundamental level, geometrically nonlinear magnetic rod formulations provide a principled framework for capturing magneto-elastic coupling; [20] offers a reduced-order yet physically useful description. In parallel, prior work on CTRs has shown that elastic instabilities, including snap-through and multi-stability, play a key role in determining accessible configurations [21, 22].

In [8] we introduced the concept of the Coaxial Sleeve Magnetic Actuator (CoSMA) which combines the fundamental operating principle of the CTR with magnetic actuation. CoSMA is designed to operate within any large, static/quasi-static background field such as those found in MRI systems and fusion-energy devices, where magnetic energy is abundant but not controllable by the operator. Indeed, any attempt at the inclusion of magnetically remnant components in such fields results in overwriting of magnetic signature [23]. The CoSMA combines magnetically soft actuating components, exploiting easy-plane alignment as actuating torque, with CTR design. This allows for a CTR, actuated by the background field of an MRI scanner, manufactured from materials orders of magnitude softer than the traditional CTR and which inherently mitigates the unsafe *snap-through* kinetic instability of these CTR designs. In this paper we introduce a numerically solved, analytical formulation for this class of magnetic CTR, combining the mechanical modeling framework of [6] with the actuation of softly magnetic components from [24]. We solve this model using both a genetic algorithm and then, to capture mechanical hysteresis, a gradient descent algorithm. We demonstrate the range of motion of the current design of the CoSMA and reconcile simulation results to experimental data.

**Fig. 1:**
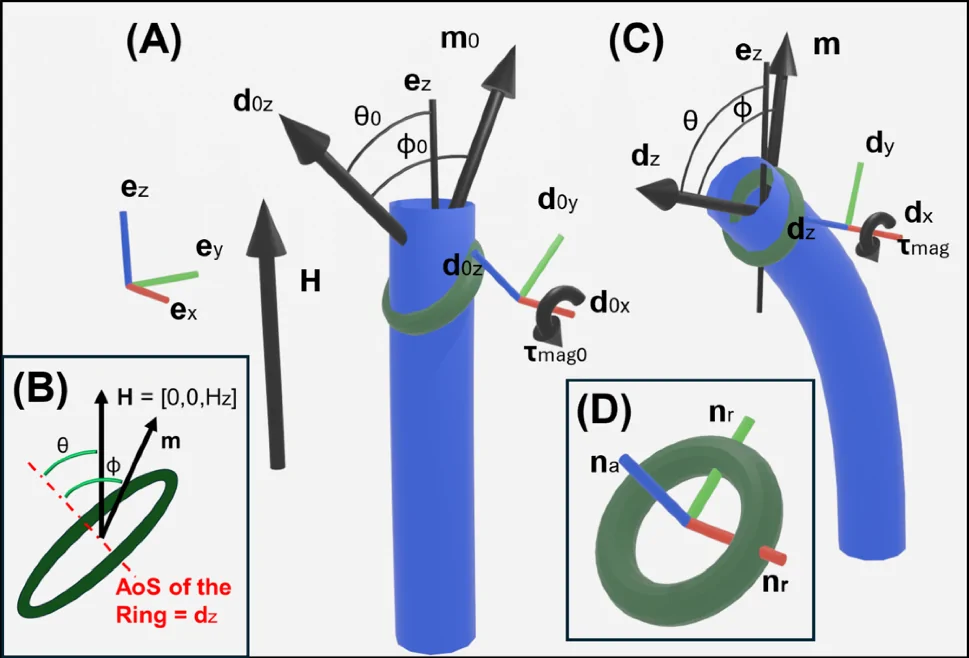
**A** The referential pose of a single sleeve of the catheter with a tip mounted iron ring. The large background field ($\boldsymbol{H}$) generates a magnetization in the ring ($\boldsymbol{m}_0$). $\phi _0$ is the angle between this induced magnetization and the background field. $\theta _0$ is the angle between the axis of symmetry (AoS) of the ring and the background field. **B** Shows a reduced image of what ϕ and θ represent in general terms. In the MRI bore $$\boldsymbol{H} \equiv \begin{bmatrix} 0, 0, H_z \end{bmatrix}$$. Consequently, if the axis of symmetry (AoS) of the ring were orthogonal to $\boldsymbol{e}_z$ (global frame Z-axis), a stable equilibrium would occur due to Eq. 6. By implanting an offset ($\theta _0$), a controllable magnetic torque ($\boldsymbol{\tau }_{mag}$) is generated. **C** The deformed pose of the same single sleeve. Magnetic torque generates elastic deformation ($\boldsymbol{u}$ in Eq. 16) changing $\theta _0$ to θ and thus $\phi _0$ to ϕ. Importantly, ϕ is not directly observable whereas θ can be reconstructed from knowledge of $\theta _0$ and $\boldsymbol{u}$. Magnetic torque is therefore a function of deformation. **D** $n_a = n_z$ is the axial demagnetization factor and $n_r = n_x = n_y$ is the radial demagnetization factor. These factors are bound by the constraint $n_a+2n_r = 1$

## Open form analytical formulation

Here we detail an analytical formulation of a concentric sleeve catheter where the deformation energy source originates from a background magnetic field, as opposed to the more commonly described formulation, where deformation energy is stored in the elasticity of a pre-bent tube.

### Single sleeve magneto-elastic balance

Any non-spherical object in a magnetic field will experience some magnetic torque associated with the “easy-plane of alignment” (See Fig. 1A). The magnitude and direction of this torque is exactly encoded for non-remnant materials and ellipsoidal geometries in [24] via the concept of *demagnetization factors*. The design considered here does not approximate to an ellipsoid in any meaningful manner and, as in [8], demagnetization factors must be determined via numerical methods. For a circular hoop with the only axis of symmetry (AoS) aligned with the local Z-axis ($\boldsymbol{d}_z$ in Fig. 1) $n_a = n_z$ is the axial demagnetization factor and $n_r = n_x = n_y$ is the radial demagnetization factor aligned with the local X- and Y-axes respectively ($\boldsymbol{d}_x, \boldsymbol{d}_y$ in Fig. 1). These factors are bound by the constraint $n_x+n_y+n_z=1$ thus, due to symmetry, $n_a+2n_r = 1$. For our homogeneous field, demagnetization factors are a function of geometry only, they are defined in a body-fixed frame and invariant to applied field direction and therefore deformation of our catheter (See Appendix 1 for more details on demagnetization factors). Furthermore, for typical MRI field strengths ($B_0 \ge 1.5\,\textrm{T}$), iron approaches its saturation magnetization ($m_s = 1.43 \times 10^6 A/m$), for higher field scanners such as ours ($B_0 \ge 7.0\,\textrm{T}$) it far exceeds saturation. Consequently, the magnetic response can be reasonably approximated as operating in the saturated regime.

From [25] and [24], for a magnetically saturated object of known $n_r,n_a$ with volume *V*, and saturation magnetization $m_s$, where ϕ is the angle between the magnetization of the object and AoS of the object, $|\boldsymbol{H}|$ is the magnitude of the background field and θ is the angle between the applied field and the AoS of the object (See Fig. 1), the magnetic energy ($P_{mag}$) is given by:

1 $$\begin{aligned}  &   P_{mag} = \frac{1}{2}\mu _0 V (n_r - n_a) m_s^2 \sin ^2(\phi )\nonumber \\    &   \quad - \mu _0 V m_s |\boldsymbol{H}| \cos (\theta - \phi ). \end{aligned}$$

The first term on the right hand side represents the anisotropic energy - that due to the difference in demagnetization factors, and the second term on the right hand side represents the Zeeman energy - the interaction of magnetization with applied field.

**Fig. 2:**
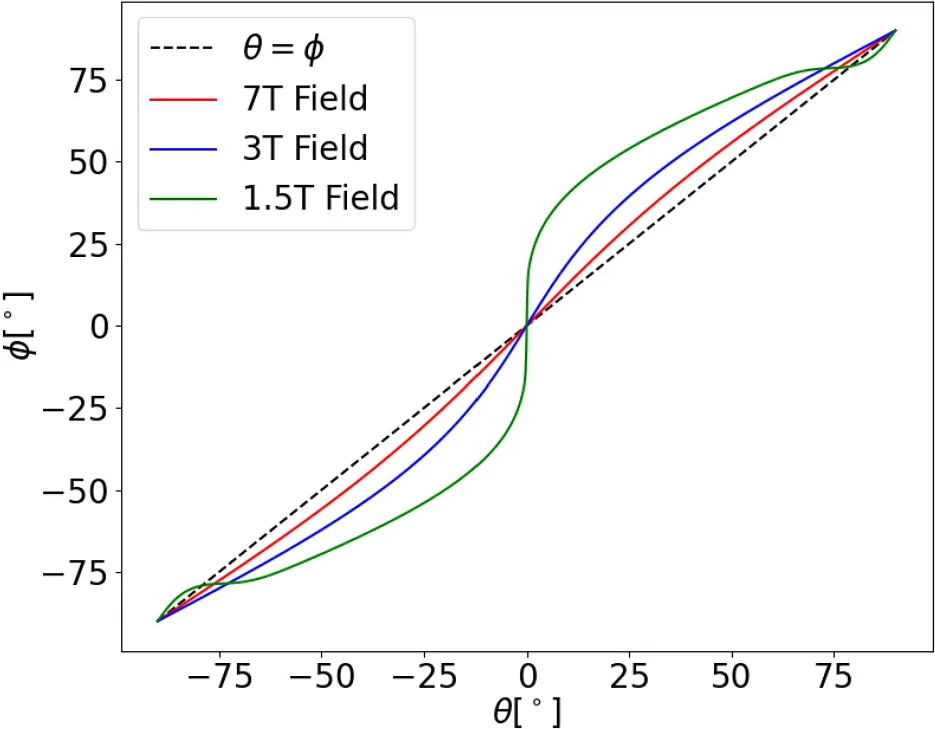
The numerical solution to Eq. 3 for $\theta \in [ -90^\circ ,90^\circ ]$. The black hatched line shows the curve $\theta =\phi$, a common assumption in high background fields. Other curves pertain to background fields ($B=\mu _0H$) in common clinical and pre-clinical MRI scanners, 7T, 3T and 1.5T. In our 7T scanner this assumption produces an RMS error of 7.7% peaking at 10.9% when $\theta =\pm 40^\circ$

Equation 1 can be minimized with respect to ϕ (for any fixed θ) to determine the relationship between ϕ and θ (See Fig. 2):

2 $$\begin{aligned}  &   \frac{\partial P_{mag}}{\partial \phi } = 0, \end{aligned}$$

3 $$\begin{aligned}  &   (n_r - n_a ) m_s \sin (2\phi ) = 2|\boldsymbol{H}| \sin (\theta - \phi ). \end{aligned}$$

The negative of the differential of Eq. 1 with respect to θ gives the magnitude of global frame magnetic torque:

4 $$\begin{aligned} |\boldsymbol{\tau }_{mag}^{global}| = -\frac{dP_{mag}}{d \theta } = -\Big (\frac{\partial P_{mag}}{\partial \theta } + \frac{\partial P_{mag}}{\partial \phi }.\frac{\partial \phi }{\partial \theta }\Big ), \end{aligned}$$

and from Eq. 2:

5 $$\begin{aligned} |\boldsymbol{\tau }_{mag}^{global}|= -\frac{\partial P_{mag}}{\partial \theta } = - \mu _0 V m_s |\boldsymbol{H}| \sin (\theta - \phi ), \end{aligned}$$

which, from Eq. 3, gives:

6 $$\begin{aligned} |\boldsymbol{\tau }_{mag}^{global}| = \frac{1}{2}\mu _0 V |n_r - n_a| m_s^2 \sin (2\phi ). \end{aligned}$$

With further reference to Fig. 1, defining $\boldsymbol{d}_{0z}$ as the referential unit vector in the direction of the symmetrical axis of the iron hoop:

7 $$\begin{aligned} \boldsymbol{d}_z = R_{L}\boldsymbol{d}_{0z}. \end{aligned}$$

$\boldsymbol{d}_z$ is the deformed unit vector in the direction of the AoS of the iron hoop where $R_{L} \in SO(3)$ is the rotation matrix of the iron hoop due to catheter deformation:

8 $$\begin{aligned} R_{L} = \exp (\boldsymbol{\gamma }_L|_X) \end{aligned}$$

where exp(.) indicates the matrix exponent and $\boldsymbol{\gamma }_L|_X$ is the skew symmetric matrix of the vector $\boldsymbol{\gamma }_L \in \mathbb {R}^3$, defined as the rotation vector of angles between referential and deformed poses at the tip (s=L):

9 $$\begin{aligned} \boldsymbol{\gamma }_L = \int _0^L \boldsymbol{u}(s)\, ds \end{aligned}$$

where $\boldsymbol{u}(s) \in \mathbb {R}^3$ is the Darboux (curvature–twist) vector, expressed in the local material frame and measured in radians per metre (${u}_x$ and ${u}_y$ represent bending, whilst ${u}_z$ represents twisting). It is noteworthy here that, whilst the assumption of constant curvature is a convenient and efficient modeling approach, it does impose a strong kinematic simplification. In any future situation where external wrenches (e.g. environmental contact) or body forces (e.g. gravity) were implemented this assumption would need to be revisited.

Global magnetic torque is the product of torque direction ($\hat{\boldsymbol{\tau }}_{mag}^{global}$) and torque magnitude ($|{\boldsymbol{\tau }}_{mag}^{global}|$, from Eq. 6). As the direction of magnetization is not directly observable, we derive the torque direction ($\hat{\boldsymbol{\tau }}_{mag}^{global}$) in terms of the unit vector of the AoS of the iron ring ($\boldsymbol{d}_z$). As $\boldsymbol{m}$ always exists in the plane containing $\hat{\boldsymbol{d}_z}$ and $\boldsymbol{H}$, and $\hat{\boldsymbol{H}} = \boldsymbol{e}_z$, torque direction can be defined as:

10 $$\begin{aligned} \hat{\boldsymbol{\tau }}_{mag}^{global} = \frac{\boldsymbol{m} \times \boldsymbol{H}}{|\boldsymbol{m} \times \boldsymbol{H}|} = \frac{\boldsymbol{d}_z \times \boldsymbol{e}_z}{|\boldsymbol{d}_z \times \boldsymbol{e}_z|} = \hat{\begin{bmatrix} \boldsymbol{d}_{z(y)} \\ -\boldsymbol{d}_{z(x)} \\ 0 \end{bmatrix}}, \end{aligned}$$

where $\boldsymbol{d}_{z(x)}$ and $\boldsymbol{d}_{z(y)}$ are the global *x* and *y* components of $\boldsymbol{d}_{z}$ respectively.

Thus, global magnetic torque can be defined as:

11 $$\begin{aligned} \boldsymbol{\tau }_{mag}^{global} = \hat{\boldsymbol{\tau }}_{mag}^{global}.|\boldsymbol{\tau }_{mag}^{global}| = c \sin (2\phi ) \hat{\begin{bmatrix} \boldsymbol{d}_{z(y)} \\ -\boldsymbol{d}_{z(x)} \\ 0 \end{bmatrix}}, \end{aligned}$$

where:

12 $$\begin{aligned} c = \frac{1}{2}\mu _0 V |n_r-n_a|m_s^2. \end{aligned}$$

Referring again to Fig. 1, θ represents the angle between the AoS of the ring ($\boldsymbol{d}_{z}$) and the background field ($\boldsymbol{e}_{z}$). The dot product of these gives:

13 $$\begin{aligned} \boldsymbol{d}_{z}.\boldsymbol{e}_{z} = |\boldsymbol{d}_{z}||\boldsymbol{e}_{z}|\cos (\theta ) = \cos (\theta ) = \boldsymbol{d}_{z(z)}. \end{aligned}$$

From [24] and Fig. 2 we know that for large applied fields $\theta \approx \phi$, thus:

14 $$\begin{aligned} \boldsymbol{\tau }_{mag}^{global} = c \sin (2\arccos (\boldsymbol{d}_{z(z)})) \hat{\begin{bmatrix} \boldsymbol{d}_{z(y)} \\ -\boldsymbol{d}_{z(x)} \\ 0 \end{bmatrix}}, \end{aligned}$$

which gives (see Appendix 3):

15 $$\begin{aligned} \boldsymbol{\tau }_{mag}^{global} = 2c\boldsymbol{d}_{z(z)} {\begin{bmatrix} \boldsymbol{d}_{z(y)} \\ -\boldsymbol{d}_{z(x)} \\ 0 \end{bmatrix}} \end{aligned}$$

This is intuitive as, for any fixed *c* (Eq. 12), global magnetic torque is purely a function of the orientation directors of the axis of symmetry of the ring. Recall from Eq. 7 that these directors are a function of curvature along the length of the catheter.

**Fig. 3:**
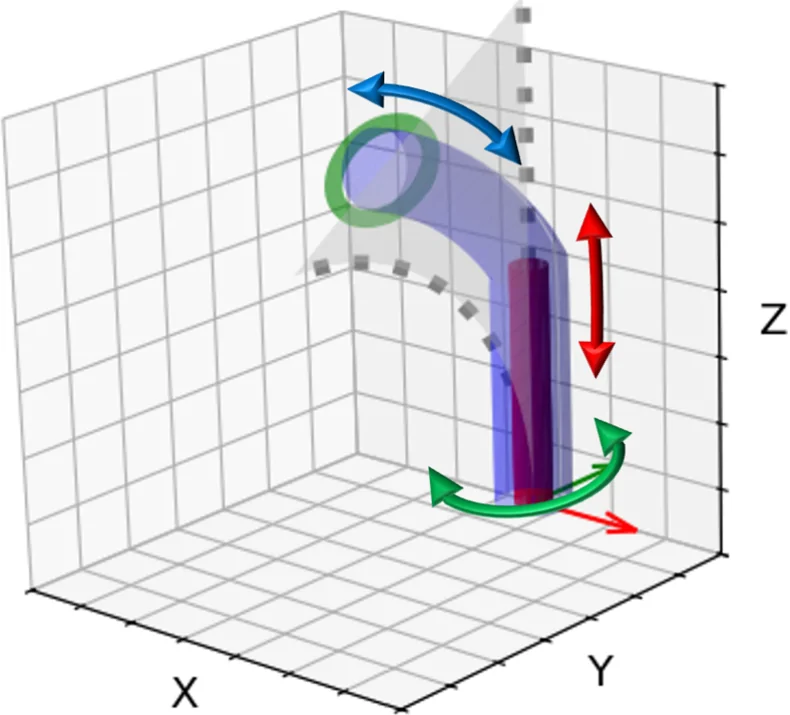
An internal rod of significantly higher bending stiffness (in red) can be inserted into Sleeve 1 (in blue) to increase range of motion (in gray). This adds a further DoF beyond the analysis presented here. The entire system is axially symmetric and can be rotated to produce a complete volumetric range of motion up to a maximum tip deformation of $\approx 80^\circ$. A demonstration of Eq. 16 formulated into the inverse Jacobian is shown in supplementary video S1 with derivation in Appendix 2

Consequently, we impose the magnetic torque as a boundary condition at the distal end. In the absence of any external wrenches along the rod length, this results in a constant internal bending moment ($\textbf{M}$) along the arc length (i.e. $d\textbf{M}/ds = 0$). Expressed in the local material frame, this corresponds to a constant moment vector, which through the constitutive relation $\textbf{M} = K\textbf{u}(s)$ corresponds to a three-dimensional constant-curvature deformation. Magnetic torque rotated into the local catheter frame, must equal elastic torque:

16 $$\begin{aligned} \boldsymbol{\tau }_{mag}^{local}(s) = R(s)^T\boldsymbol{\tau }_{mag}^{global} = K \boldsymbol{u}(s), \end{aligned}$$

where *R*(*s*) is the rotation matrix formulated as in Eq. 8 but calculated at any point *s* along the sleeve. *K* is the diagonal 3D stiffness matrix (in $Nm^2$) with the first two elements representing bending stiffness and the third element representing twisting stiffness [26]. For isotropic materials these stiffness parameters would traditionally be represented by the product of Elastic modulus and Second moment of area (EI) for bending stiffness and the product of Shear modulus and Polar moment of inertia (GJ) for twisting stiffness. For anisotropic material construction this parameterization loses meaning but we preserve the nomenclature:

17 $$\begin{aligned} K = diag(EI,EI,GJ)= diag(K_x,K_y,K_z). \end{aligned}$$

For a twist free elastic rod subject to a point torque at the tip, the bending curvatures ${{u}}_{x}$ and ${{u}}_{y}$ are constant with respect to *s*, i.e. $\dot{{{u}}}_{x} = \dot{{{u}}}_{y} = 0$ (the dot indicates differential w.r.t. s) and the twisting curvature ${u}_{z}=0$. Even this simple case (Fig. 1) has no closed-form solution due to the inter-dependency of torque and curvature but a numerical solution to the torque balance given in Eq. 16 for a single sleeve is easily and rapidly achievable. Figure 3 shows a solution to Eq. 16 where K experiences a step change due to an internal rod of significantly higher bending stiffness being inserted within Sleeve 1. This produces a 2 DoF system with the range of motion shaded in gray. Clearly, rotation of the system about the global Z-axis would produce a 3 DoF system with a complete volumetric range of motion. Exploration of this extra DoF lies beyond the scope of this work but this information is included to illustrate that the system we present is capable of the full range of motion. In Appendix 2 we derive the Jacobian from the solution of Eq. 16 and the supporting video S1 shows the inverse Jacobian controller in simulation.

**Fig. 4:**
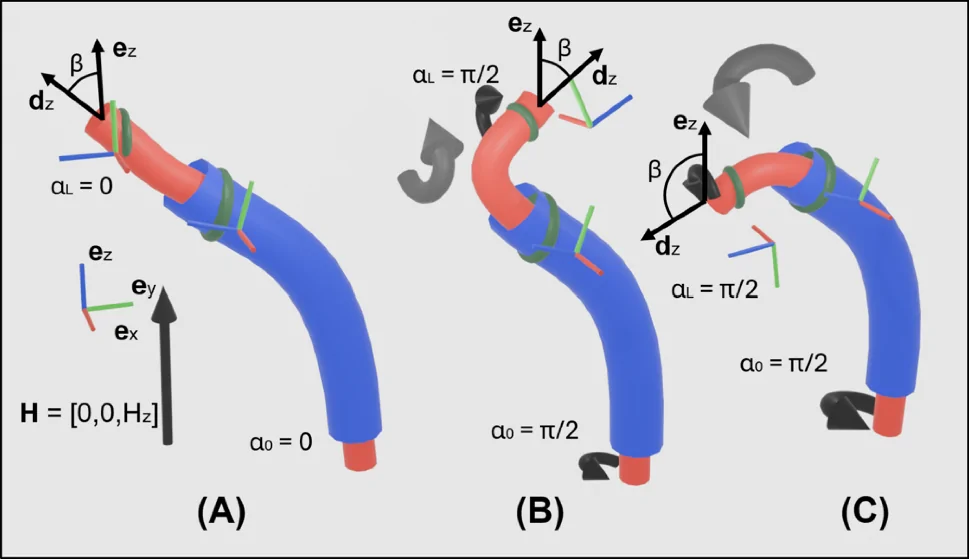
The outer sleeve (Blue) is base constrained whilst the inner sleeve (Red) is rotated at the base ($\alpha _0$). This rotation maps nonlinearly to the rotation of the tip ($\alpha _L$). Initially $\alpha _L$ lags $\alpha _0$, then at some critical point $\alpha _L$ “snaps” forward. This snapping instability is mitigated by using an extremely low stiffness ratio, braided sleeve. Whether the inner sleeve snaps into the forward facing configuration or the backward facing configuration is dependent on the length of unconstrained inner sleeve

### Nested pair of sleeves

**Fig. 5:**
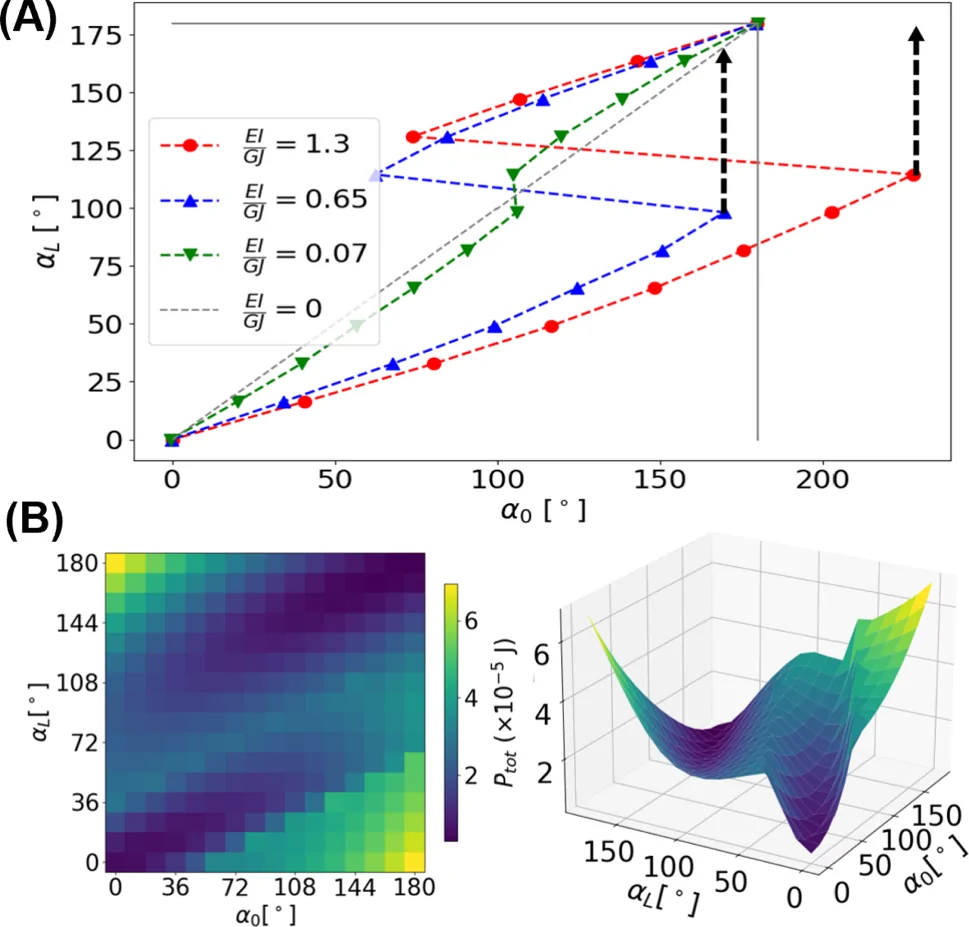
(**A**) $\alpha _0$ against $\alpha _L$ according to the Genetic Algorithm solution of Eq. 25 for three different stiffness ratios. In red, $\frac{EI}{GJ}=1.3$ represents isotropic construction, comparable to conventional CTRs. In blue, $\frac{EI}{GJ}=0.65$ represents the numbers presented in the patent for improving this stiffness ratio in [27]. In green, $\frac{EI}{GJ}=0.07$ represents our braided nylon construction. The snapping instability, illustrated here by the black arrows, is well reported for conventional CTRs. It can be seen that without optimized anisotropic construction our magnetic system would be so unstable it would lose all functionality, snapping straight through the higher energy deformations shown in Fig. 4. With the mechanical stability afforded by our low stiffness ratio design, the snapping region diminishes sufficiently that unwanted twisting is no longer a major operational consideration. There is still, however, a prohibited operating region which we explore in the next section. (**B**) A sample energy landscape of the CoSMA as $\alpha _0$ is Z-rotated. Height indicates total system energy. Two low energy valleys occur when $\alpha _0<\alpha _L$ giving $\dot{\alpha }>0$ and when $\alpha _0>\alpha _L$ giving $\dot{\alpha }<0$. The critical $\alpha _0$ at which this energy discharge occurs is a function of the system design (referential ring poses and ring geometries, sleeve material and geometric properties) and the catheter pose, particularly the lengths of both sleeves

Due to the presence of sin(2ϕ) in Eq. 6, torque decays to zero every 90$^\circ$. This is physically evident when we consider that as we rotate a slender object in $H_0$ the induced magnetization will flip every 180$^\circ$ - every time ϕ passes through zero. This stands in contrast to the full 360$^\circ$ range of motion available when magnetically remnant bodies are placed in relatively low actuating fields (e.g. [16]). Due to this significantly different operating principle, any single magnetic body can only generate catheter deformation up to but not exceeding 90$^\circ$. As such, here we employ the concentric sleeve approach to embed a second magnetic body with a distinct geometric arrangement (varying referential ring alignment). Via rotation and translation of the two sleeves relative to each other it is possible to generate catheter deformation in excess of 90$^\circ$.

For consistency with earlier CTR work, we define the outer sleeve (blue in Fig. 4) as sleeve 1, and the inner sleeve (red in Fig. 4) as sleeve 2. The key constraint governing a concentric sleeve pair is that, in the overlapping region ($s \le L_1$) they must share the same location but not necessarily orientation. Clearly, the tangent vector must be coincident, but the normal (and bi-normal) vectors of sleeves 1 and 2 can rotate with respect to each other. This twisting angle about the local tangent is defined as α and, furthermore, due to Eq. 14, there is zero twist in sleeve 1 ($\alpha _1=0$). Clearly, α only exists in sleeve 2 and is defined as the integral of the twist rate:

18 $$\begin{aligned} \alpha _{} = \int {u}_{2z} ds = \boldsymbol{\gamma }_{2z}. \end{aligned}$$

Likewise, ${u}_{2z}$ = $\dot{\alpha }_{}$ (The over-dot denotes differentiation with respect to arc length *s*) where, due to the application of a point torque at the tip:

19 $$\begin{aligned} \dot{\alpha }= \frac{\alpha _L-\alpha _0}{L}, \end{aligned}$$

is a constant. Here $\alpha _0$ is the z-rotation of the base of sleeve 2 relative to the pose defined as that in which both AoS’s of the two ferrous rings lie in the YZ plane ($\alpha _0=0$, Fig. 4A). $\alpha _L$ is the local Z rotation of the tip of sleeve 2. As shown in Fig. 5, manual rotation of the base ($\alpha _0$) maps non-linearly to tip rotation ($\alpha _L$). Initially $\alpha _L$ lags $\alpha _0$, then at some critical point $\alpha _L$ “snaps” forward (for a catheter of infinite twisting stiffness this relationship would become linear).

The system can be characterized in three distinct regions. Sleeve 1, in its own local frame, with constant curvature in x- and y-, and zero curvature in z-:

20 $$\begin{aligned} \dot{{{u}}}_{1x} = \dot{{{u}}}_{1y} = 0, {u}_{1z}=0. \end{aligned}$$

Sleeve 2, in its own local frame, exhibits two constant curvature regions (see Fig. 4), the unconstrained region beyond sleeve 1 ($s>L_1$) with constant curvature about all axes:

21 $$\begin{aligned} \dot{{{u}}}_{2x}|_{s>L_1} = \dot{{{u}}}_{2y}|_{s>L_1} = 0, {{u}}_{2z} = \dot{\alpha }, \end{aligned}$$

and the region constrained by sleeve 1 ($s \le L_1$). This region exhibits the same twisting curvature as the distal region of sleeve 2 but bending curvatures subject to the compatibility equation [6] enforcing the coincidence of sleeve center-lines with sleeve 1 (see Appendix 4):

22 $$\begin{aligned}  &   \boldsymbol{u}_{1} = R_z(\alpha ) \boldsymbol{u}_{2} - \dot{\alpha } \boldsymbol{e}_z, \end{aligned}$$

23 $$\begin{aligned}  &   \dot{{{u}}}_{2x}|_{s\le L_1} = \dot{{{u}}}_{2y}|_{s\le L_1} = 0, {{u}}_{2z} = \dot{\alpha }. \end{aligned}$$

Here, $R_z(\alpha )$ is the rotation matrix of α radians about the local Z-axis (which is common for both sleeves) and $\boldsymbol{e}_z = [0,0,1]^T$.

In this, more complex, two-sleeve arrangement, the torque balance in Eq. 16 is prone to unstable equilibria and suffers from solution multiplicity. Even in the case that carefully selected optimization constraints mitigate these issues, Eq. 16 would need to be optimized on the 3D torque variable incurring an exponential increase in computational cost. Consequently, we adopt global system energy minimization.

**Fig. 6:**
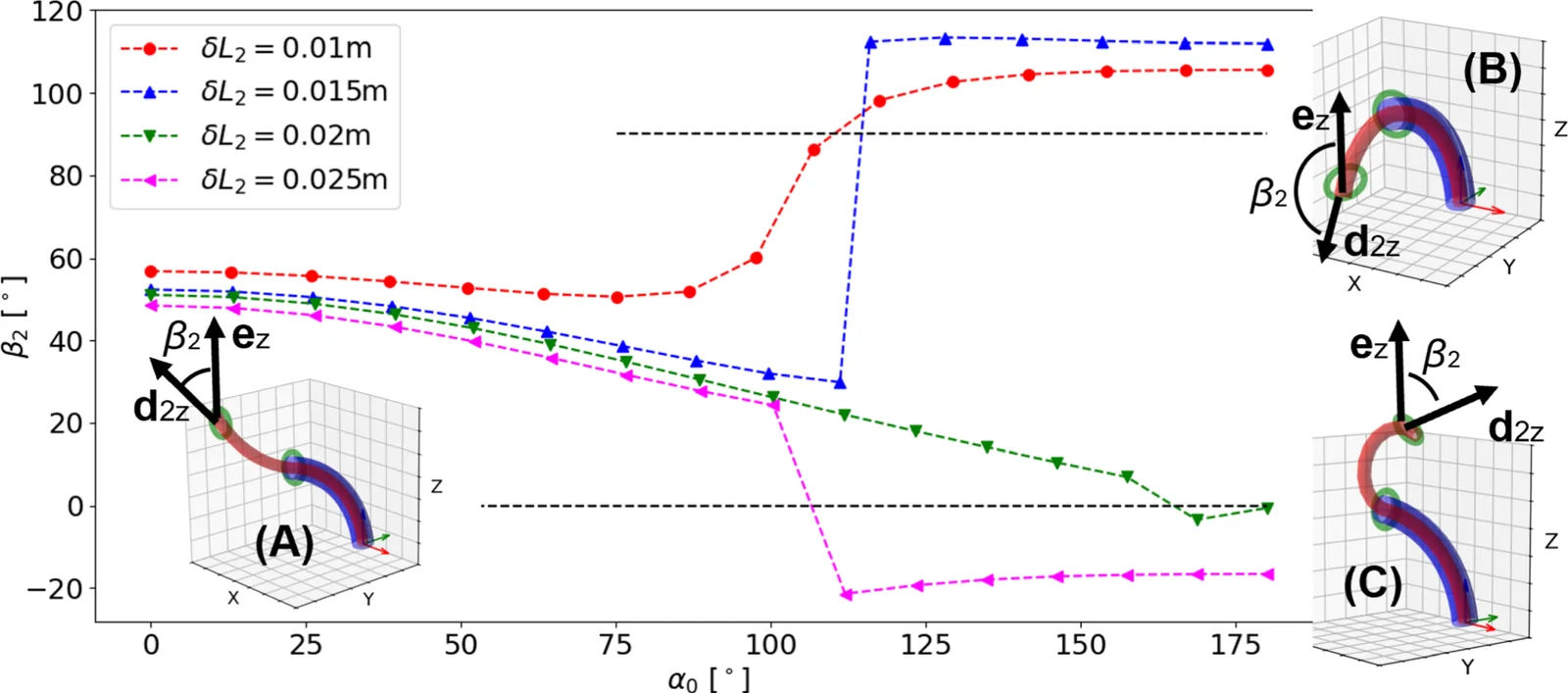
$\beta _2$ against $\alpha _0$ for a range of $\delta L_2$ values ($\delta L_2 = L_2-L_1 \in [0.01,\; 0.025]\,\text {m}$). The tip angle of sleeve 2 ($\beta _2$) starts in the lowest energy configuration (illustrated in **(A)**). The base of sleeve 2 is rotated ($\alpha _0$) through 180$^\circ$ causing the system to flip into either the backward facing configuration **(B)** or the sigmoidal configuration **(C)**. The configuration which the system selects is a function of the free length ($\delta L_2$) when the base rotation is performed - shorter $\delta L_2$ results in backward facing deformation, longer in sigmoidal deformation

For any set of 3D curvatures, $\phi _i$ can be determined for the *i*’th ring, Eq. 1 then gives magnetic energy which can be summed over the two rings. Elastic strain energy in the *i*’th sleeve is given by [28]:

24 $$\begin{aligned} P_{ela} = \frac{1}{2} \int _{0}^{L} [K_{ix}\boldsymbol{{u}}_{ix}^{2} + K_{iy}\boldsymbol{{u}}_{iy}^{2} + K_{iz}\boldsymbol{{u}}_{iz}^{2}]\, ds = \frac{1}{2} \int _{0}^{L} [\boldsymbol{u}_i^T K_i \boldsymbol{u}_i] ds \end{aligned}$$

which can also be summed over both sleeves. The minimum system energy is thus defined as the set of sleeve curvatures which minimize $P_{ela}+P_{mag}$:25where $\phi _i$ can be determined from Eq. 3 which relies on determination of $\theta _i$ from:

26 $$\begin{aligned} \boldsymbol{d}_{z} = \exp (\int _0^L \boldsymbol{u}(s)\, ds|_X)\boldsymbol{d}_{0z}. \end{aligned}$$

The optimization variables ($\boldsymbol{u}_i$) and integration limits ($L_i$) are not immediately intuitive so we clarify here. For sleeve 1, twist is assumed zero (no friction between sleeves) so, $\boldsymbol{u}_1 \in \mathbb {R}^2$. For sleeve 2, twisting deformation (α) is constant over the interval $[0, L_2]$, i.e. the full length. Bending deformation $\boldsymbol{u}_2$ over the interval $[0, L_1]$ (the constrained region) is entirely dependent on $\boldsymbol{u}_1$ and twist (α) so is zero DoF (Eq. 22). Bending deformation $\boldsymbol{u}_2$ is then constant over the interval $[L_1, L_2]$, thus $\boldsymbol{u}_2 \in \mathbb {R}^3$.

This energy minimization gives a complete representation of deformation with ${\theta }$ and ${\phi }$ included as separate, dependent variables. Eq. 25 is a composite of transcendental functions meaning it cannot be solved analytically. In real world terms, the magnetic torque is a sinusoidal function of tip pose, which is the integral of the elastic deformation along the catheter’s length. Under magnetic actuation, the torque calculation is dependent on knowledge of the full three-dimensional deformation. Consequently, Eq. 25 has been solved in the scipy genetic algorithm (GA) function (*scipy.optimize.differential_evolution*).

## Synthetic results

A catheter arrangement identical to the outer two sleeves described in [8] was modeled. Fabricated from braided sleeves with diameters of $d_1 = 3.0\,\text {mm}$ and $d_2 = 1.5\,\text {mm}$, the structure is characterized by stiffness metrics $EI_1=1.6\times 10^{-6}\,\text {N}\,\text {m}^2$, $GJ_1=1.9\times 10^{-5}\,\text {N}\,\text {m}^2$, $EI_2=1.0\times 10^{-6}\,\text {N}\,\text {m}^2$, and $GJ_2=1.4\times 10^{-5}\,\text {N}\,\text {m}^2$. For the iron ring components ($d_{\text {minor}}=0.25\,\text {mm}$), the major diameters are defined as $4.0\,\text {mm}$ and $2.1\,\text {mm}$ for sleeves 1 and 2, respectively, with demagnetization factors numerically calculated via FEM as $n_{a1} = 0.95$ and $n_{a2} = 0.86$ (see Appendix 1). Ring attachment was modeled at reference orientation angles of $\theta _1=80^\circ$ and $\theta _2=45^\circ$ [8]. The magnetic environment is dictated by a saturation magnetization of $m_s = 1.43 \times 10^6\,\text {A/m}$ under a uniform $B_0=7\,\text {T}$ background field produced by a preclinical Bruker 7T BioSpec 70/20 MRI system. For more details refer to the supporting material of [8].

In Fig. 5, the energy minimization (Eq. 25) is performed at a series of fixed z-orientations of sleeve 2. For high stiffness ratio (including isotropic) material designs, any input value of $\alpha _0$ suffers from solution multiplicity such that the genetic algorithm (GA) must be solved for $\alpha _{0}$ for a known $\alpha _{L}$ - any input value of $\alpha _{L}$ gives a unique solution value of $\alpha _{0}$ whilst the reverse is not true. The GA converges for each data point in the order of 10 s (10 randomly selected configurations, runtime = 6.29±0.95s). As the base is rotated ($\alpha _0$ increased), $\dot{\alpha }$ also increases causing a build-up of total system energy. Near the transition point the energy barrier separating the two configurations decreases. At some critical $\alpha _0$, the energy barrier vanishes and the system releases stored energy via the snap-through instability as it transitions through $\dot{\alpha }=0$ to the new configuration. This process gives rise to the bistable (hysteretic) response of the entire system and can be leveraged to increase overall reachable space of the catheter. The impact of the ratio of bending to twisting stiffness (*EI*/*GJ*) on system stability is clearly seen as a reduced snapped through region, illustrated by the black hatched arrows in Fig. 5.

**Fig. 7:**
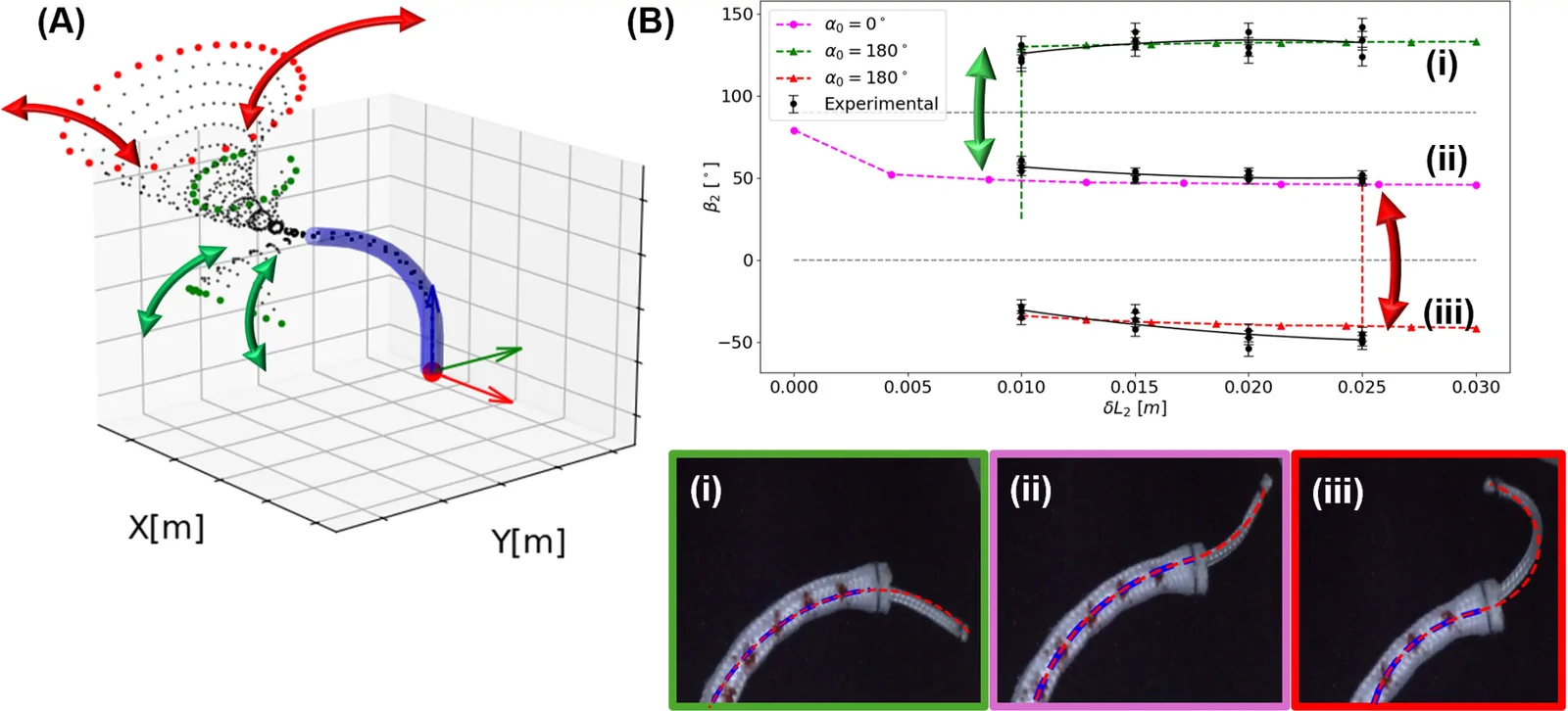
(A) Range of motion plot based on the gradient descent solution of Eq. 25. (B) Numerical versus Experimental results. The initial pose at insertion follows the curve in magenta (ii). $\alpha _0$ is rotated at $\delta L_2=0.01m$ to create the backward facing pose - the green curve (i). $\alpha _0$ is rotated at $\delta L_2=0.025m$ to create the sigmoidal pose - the red curve (iii). Post rotation, sleeve 2 can be inserted and retracted without losing shape. Simulation results are superimposed onto experimental images in (i),(ii) and (iii). Underlying experimental deformations shown in supplementary video S1

Importantly, for our stabilized - low stiffness ratio - design, the solution multiplicity described above disappears. One direct, and useful, consequence of this physical behavior is that we can use a gradient descent algorithm (*scipy.optimize.minimize*), as opposed to a GA, and optimize on $\alpha _0$ instead of $\alpha _L$. This converges in 3% of the run-time of the GA (128 configurations, runtime = 0.126±0.074 s) but, more importantly, allows us to use the previous result as the initial conditions for the following run. Critically, this means we can capture mechanical hysteresis within our simulation.

The control inputs in our formulation are the base rotation of sleeve 2, $\alpha _0$, and the unconstrained length of sleeve 2 ($\delta L_2$). To capture hysteresis, we solve the energy minimization sequentially over increments of $\alpha _0$, using the solution at the previous step as the initial condition for the next. This continuation strategy enforces convergence to a nearby local minimum, thereby allowing the solver to track a specific equilibrium branch through configuration space. In this way, path-dependent behavior (mechanical hysteresis) naturally emerges, as different solution branches are followed depending on the direction and history of the input.

Adopting the gradient descent approach, Fig. 6 shows the effect of base rotation ($\alpha _0$) on the tip angle of sleeve 2 ($\beta _2$). The tip angle of sleeve 2 starts in the lowest energy configuration (A). The base of sleeve 2 is rotated ($\alpha _0$) through 180$^\circ$ causing the system to flip into either the backward facing configuration (B) or the sigmoidal configuration (C). The free length ($\delta L_2$) when the base rotation is performed determines the resultant pose. If sleeve 2 is rotated when $\delta L_2 < 20mm$ it will flip into the backward facing pose. If sleeve 2 is rotated when $\delta L_2 > 20mm$ it will flip into the sigmoidal pose. Once the pose is set, either sigmoidal or backward facing, sleeve 2 is locked in and can be inserted and retracted (as in Fig. 7) maintaining this shape. Consequently, we can observe two different poses with identical base configurations and different historical paths. This bistability has been demonstrated to expand the range of motion of the system [8] but here we encode, via simulation, the rules by which the various poses can be attained. It is noteworthy at this point that we have only considered run-time variables in this analysis. The objective of this article is to capture the behavior of the CoSMA in simulation, not to optimize design. The critical $\alpha _0$ and $\delta L_2$ at which the energy discharge occurs are also a function of (1) referential ring poses ($\theta _{0}$), (2) ring demagnetization factors ($n_{r}$) and (3) sleeve material and geometric properties ($\frac{EI}{GJ}$)). These parameters pertain to system design which is beyond the scope of this work. Furthermore, the range of motion can be significantly enhanced by leveraging the linear variable stiffness shown in Fig. 3, this would, again, affect the critical location of energy discharge. These variations can all be captured by the model presented but also lie beyond the scope of this work.

## Experimental results

The fabrication process followed the protocol detailed in [8]. Sleeve 1 consisted of a 50 mm long, 3 mm diameter braided nylon sleeve (Lead-Weight Tape 100 g, Merrick & Day, UK) outfitted with a single orthogonal winding of 0.25 mm diameter iron wire (Merck KGaA, Germany), secured via adhesive. For sleeve 2, the same iron wire was wound orthogonally around a temporary 2.1 mm diameter mandrel to guarantee a circular geometry, preventing any shape-induced alignment bias. This prefabricated ring was then affixed at a $45^\circ$ angle to a 75 mm long, 1.5 mm diameter nylon braid (James Lever 1856 Ltd, UK). To facilitate manual control from outside the bore of the 7T pre-clinical MRI scanner (Bruker BioSpec 70/20), both sleeves were glued to 600 mm lengths of concentric fiberglass cabling before being assembled in a concentric tube configuration. Finally, a 3.9 mm diameter, MRI-compatible 720P endoscopic camera (Shenzhen Licam Technology Co., Ltd.) was integrated onto a sealed, custom 3D-printed housing equipped with an insertion tube for the catheter. For more details refer to the supporting material of [8].

A custom Python algorithm applied the atan2 formula to a geometrically corrected 2D video of the catheter, against a checkerboard background, to determine the tip angle $\beta _2$ through the known direction of $B_0$ and the catheter tip on the selected frame. Results shown in Fig. 7 for three experimental repeats show an overall RMS tip error of $4.4^\circ$ (10%) which can be subdivided into $2.7^\circ$ (5%) for the initial pose, $9.7^\circ$ (23%) for the backward facing pose and $0.8^\circ$ (1%) for the sigmoidal pose. For full body shape reconstruction, screen-grabs were taken at the planar data-points shown in Fig. 7B. These images had their centerlines extracted (binary thresholding and skeletonization found the longest 1D path) followed by Gaussian smoothing ($\sigma = 10\%$). The final centerline coordinates were normalized to known catheter length and RMS error was calculated against simulation data. Total RMSE was 1.36±0.47mm which is, normalized over catheter length, $2.47\pm 0.93\%$. Sample simulation results are shown superimposed onto experimental images in Fig. 7B(i),(ii) and (iii). The majority of this error is likely attributable to the manual and unregulated manufacturing process. The formalization and automation of this process remains an essential future work.

## Conclusions and future work

In this paper, we introduced an analytical model for a high-field magnetic CTR and solved it to capture mechanical hysteresis and bistability. We characterized the range of motion of the current CoSMA design and reconciled simulation results with experimental data. This article hasn’t considered the potential that the ferrous rings could be a different shape, size or, critically, could be fitted in different referential poses ($\theta _0$). These design considerations would have profound implications on the behavior of the CoSMA but lie beyond the scope of this work. The article is also dedicated almost entirely to analysis of the behavior of sleeve 2. The insertion of an inner stiffener of variable length, entirely encapsulated within sleeve 1 (Fig. 3, Appendix 2 and supporting video), would change results (but not the modeling fundamentals), as would inserting the entire system at an angle to $B_0$. These extra DoFs add clinical legitimacy to the design of the CoSMA, although developing a stable model to accommodate them remains an outstanding work. We intend to continue development in this direction.

The long-term objective of the CoSMA, specifically, is a miniature, self-sensing robot leveraging the imaging capability of the MRI scanner. Torque based actuation allows the use of significantly less ferrous volume compared to the more typical force based approaches (e.g. [29]) reducing the risk of unwanted actuation. Additionally, the use of iron (a naturally occurring bio-element), as opposed to NdFeB (a corrosive rare-earth alloy that can release cytotoxins) can help CoSMA to transcend the current state of the art in safety and bio-compatibility terms. Manufacturing improvements are required - lower diameter sleeves and digitizing the addition of the ferrous rings, either via embedding during sleeve manufacture or printing onto the sleeves. Furthermore, delicate anatomy must be protected from the rigid and potentially sharp edges of the metal rings. Laser cut and burred rings encapsulated by a thin layer of high stiffness elastomer (e.g. PDMS) can mitigate these issues.

Imaging/sensing is another large area of ongoing work which remains untouched by this contribution - Advancements in this area will address current challenges in experimental reconciliation imposed by the challenges of obtaining visual data within the MRI bore. The work presented here establishes a general modeling paradigm for a high-field magnetic CTR, providing a foundation that can be extended through future advances in design, fabrication, and sensing. While the present results focus on open-loop, path-dependent shape selection, the proposed model provides a foundation for future control development. In particular, the observed bistability indicates that the system cannot be fully described by a static input–output relationship. By capturing these effects, the model enables identification of multiple equilibria and transition thresholds between stable states, critical for reliable operation. This suggests that effective control strategies will need to incorporate state awareness or history-dependent formulations, such as branch tracking or model-based predictive control. In terms of achievable frequency of feedback control, our (non time optimized) model operates at a comparable rate (≈130 ms per step) to previously reported imaging speeds in real-time tracking for interventional MRI (≈150 ms [30]). This is not fast enough for dynamic control but sufficient for the quasi-static pose guidance which is typical in this field.

## Supplementary Information

Below is the link to the electronic supplementary material.

Supplementary file 1 (mp4 165515 KB)

## Data Availability

No datasets were generated or analysed during the current study.

## Appendix

### A1. Magneto-static finite element model

The numerical determination of demagnetization factors is covered in some detail in the supporting material of [8]. A summary of this is included here for completeness. A Finite Element Model (FEM) was constructed in the magneto-statics module of COMSOL multiphysics v6 (COMSOL AB, Stockholm, Sweden). Defining *U* as the scalar magnetic potential (in *A*) such that total field $\boldsymbol{H} = - \nabla U$ (in *A*/*m*). Because $\boldsymbol{H} = \boldsymbol{B}/\mu _{0} - \boldsymbol{m}$ and $\nabla . \boldsymbol{B} == 0$ (where $\boldsymbol{B}$ is the magnetic flux density in *T* and $\boldsymbol{m}$ is the magnetic moment in *A*/*m*), $\nabla ^2U = \nabla \boldsymbol{m}$ within the material domain and $\nabla ^2U = 0$ outside the material domain (where $\boldsymbol{m}=0$). Numerically solving these second order differential equations over a 500,000 node mesh generates a solution for the point-wise magnetization. This can be volumetrically integrated to determine the bulk magnetization vector ($\boldsymbol{M}$) which, given a known applied field, tells us the demagnetization factors from $\textbf{M} = \boldsymbol{\chi _{a}}\boldsymbol{H}$.

### A2. Derivation of Jacobian from Fig. 3

Considering the simplified planar system with a variable length stiffener (Fig. 3). The joint space vector $q \in \mathbb {R}^2$, with the two inputs being $s_1$, the insertion length of the curved tube, and $s_2$, the insertion length of a rigid inner tube which must always be straight and where $s_2 \le s_1$. Under this model $\textbf{u} = [u_x,0,0]$ and from Eq. 16:

27 $$\begin{aligned} u_x = \frac{c}{K} \sin \big (2(\theta _0+(s_1-s_2)u_x)\big ), \end{aligned}$$

which is zero when $s \le s_2$ and constant when $s>s_2$. Equation 27 is solved numerically at every iteration and the results input to the Jacobian inverse.

The position vector is:

28 $$\begin{aligned} \boldsymbol{r}(s_{1},s_{2}) = \begin{bmatrix} 0 \\ 0 \\ s_{2} \end{bmatrix} + \frac{1}{u_x} \begin{bmatrix} 0 \\ 1 - \cos \!\big ((s_{1}-s_{2})u_x\big ) \\ \sin \big ((s_{1}-s_{2})u_x\big ) \end{bmatrix}. \end{aligned}$$

The orientation is:

29 $$\begin{aligned} \boldsymbol{\omega }(s) = \begin{bmatrix} (s_1-s_2)u_x \\ 0 \\ 0 \end{bmatrix}, \end{aligned}$$

and the Jacobian:

30 $$\begin{aligned} J(s_1,s_2) = \begin{bmatrix} \displaystyle \frac{\partial \boldsymbol{r}}{\partial s_1} &  \displaystyle \frac{\partial \boldsymbol{r}}{\partial s_2} \\ \displaystyle \frac{\partial \boldsymbol{\omega }}{\partial s_1} &  \displaystyle \frac{\partial \boldsymbol{\omega }}{\partial s_2} \end{bmatrix} \end{aligned}$$

Gives:

31 $$\begin{aligned} J(s_1,s_2) = \begin{bmatrix} 0 &  0 \\ \sin ((s_1-s_2)u_x) &  -\sin ((s_1-s_2)u_x) \\ \cos ((s_1-s_2)u_x) &  1 - \cos ((s_1-s_2)u_x) \\ u_x &  -u_x \\ 0 &  0 \\ 0 &  0 \end{bmatrix}. \nonumber \\ \end{aligned}$$

### A3. Derivation of Eq. 15

$$\begin{aligned} \boldsymbol{\tau }_{mag}^{global} = c\cdot \sin (2\arccos (\boldsymbol{d}_{z(z)}))\cdot \hat{\begin{bmatrix} \boldsymbol{d}_{z(y)} \\ -\boldsymbol{d}_{z(x)} \\ 0 \end{bmatrix}} \end{aligned}$$

$$\begin{aligned} \sin \!\left( 2\arccos \!\left( \boldsymbol{d}_{zz}\right) \right)&= 2 \sin \!\left( \arccos \!\left( \boldsymbol{d}_{zz}\right) \right) \cos \!\left( \arccos \!\left( \boldsymbol{d}_{zz}\right) \right) \\&= 2\,\boldsymbol{d}_{zz}\sqrt{1-\boldsymbol{d}_{zz}^{2}} \end{aligned}$$

$$\begin{aligned} \hat{\begin{bmatrix} \boldsymbol{d}_{z(y)} \\ -\boldsymbol{d}_{z(x)} \\ 0 \end{bmatrix}}&= \frac{1}{\sqrt{\boldsymbol{d}_{z(y)}^2 + \boldsymbol{d}_{z(x)}^2}} \begin{bmatrix} \boldsymbol{d}_{z(y)} \\ -\boldsymbol{d}_{z(x)} \\ 0 \end{bmatrix} \\ \\&= \frac{1}{\sqrt{1-\boldsymbol{d}_{z(z)}^2}} \begin{bmatrix} \boldsymbol{d}_{z(y)} \\ -\boldsymbol{d}_{z(x)} \\ 0 \end{bmatrix} \\ \end{aligned}$$

32 $$\begin{aligned} \boldsymbol{\tau }_{mag}^{global}&= c \left( 2\boldsymbol{d}_{z(z)}\sqrt{1-\boldsymbol{d}_{z(z)}^2}\right) \quad \cdot \left( \frac{1}{\sqrt{1-\boldsymbol{d}_{z(z)}^2}} \begin{bmatrix} \boldsymbol{d}_{z(y)} \\ -\boldsymbol{d}_{z(x)} \\ 0 \end{bmatrix}\right) \nonumber \\&= 2c \cdot \boldsymbol{d}_{z(z)} \begin{bmatrix} \boldsymbol{d}_{z(y)} \\ -\boldsymbol{d}_{z(x)} \\ 0 \end{bmatrix} \end{aligned}$$

### A4. Center-line compatibility equation

For each sleeve, we attach a material frame $R_i(s) \in SO(3)$ along the backbone such that, as *s* moves along the rod, the local frame rotates with curvature (angular rate) $\boldsymbol{u}_i(s)$, which encodes bending and twisting:

33 $$\begin{aligned} \frac{\partial R_i(s)}{\partial s} = \dot{R_i}(s) = R_i(s) (\boldsymbol{u}_i|_X). \end{aligned}$$

These frames differ only by a relative rotation about the local z-axis:

34 $$\begin{aligned} R_2(s)=R_1(s) R_z(\alpha ). \end{aligned}$$

Differentiating both sides with respect to s:

35 $$\begin{aligned}  &   \dot{R}_2(s)= \dot{R}_1(s) R_z(\alpha ) + R_1(s) \dot{R}_z(\alpha ), \end{aligned}$$

36 $$\begin{aligned}  &   R_1(s) R_z(\alpha ) (\boldsymbol{u}_2|_X) = R_1(s) (\boldsymbol{u}_1|_X) R_z(\alpha ) + R_1(s) \dot{R}_z(\alpha ). \nonumber \\ \end{aligned}$$

Left-multiplying by $R_1^{-1}(s)$ and right-multiplying by $R_z^T(\alpha )$:

37 $$\begin{aligned} R_z(\alpha ) (\boldsymbol{u}_2|_X) R_z^T(\alpha ) = \boldsymbol{u}_1|_X + \dot{R}_z(\alpha ) R_z^T(\alpha ). \end{aligned}$$

From the identity [31]:

38 $$\begin{aligned} \dot{R}_z(\alpha ) R_z^T(\alpha ) = \boldsymbol{\omega }|_X, \end{aligned}$$

where, for rotation purely about the local tangent:

39 $$\begin{aligned} \boldsymbol{\omega } = \dot{\alpha } \boldsymbol{e}_z, \end{aligned}$$

and from the identity [31]:

40 $$\begin{aligned} R (\boldsymbol{u}|_X) R^T = (R\boldsymbol{u})|_X, \end{aligned}$$

gives:

41 $$\begin{aligned} \boldsymbol{u}_1|_X = (R_z(\alpha ) \boldsymbol{u}_2)|_X - (\dot{\alpha } \boldsymbol{e}_z)|_X, \end{aligned}$$

Which, removing the skew operator throughout leaves:

42 $$\begin{aligned} \boldsymbol{u}_1 = R_z(\alpha ) \boldsymbol{u}_2 - \dot{\alpha } \boldsymbol{e}_z, \end{aligned}$$

## List of symbols

$\boldsymbol{e}_x, \boldsymbol{e}_y, \boldsymbol{e}_z$: Global frame, Cartesian unit vectors $\in \mathbb {R}^3$ (−)
$\boldsymbol{d}_x, \boldsymbol{d}_y, \boldsymbol{d}_z$: Local frame, Cartesian unit vectors $\in \mathbb {R}^3$ (−)
$n_x, n_y, n_z$: Cartesian demagnetization factors (−)
$n_a, n_r$: Axial and radial demagnetization factors (−)
ϕ: Angle between magnetization and axis-of-symmetry (rad)
θ: Angle between applied field and axis-of-symmetry (rad)
$$\boldsymbol{H}$$: Background field $$\in \mathbb {R}^3$$ (A m$$^{-1}$$)
$$\boldsymbol{m}$$: Magnetization $$\in \mathbb {R}^3$$ (A m$$^{-1}$$)
$\mu _0$: Magnetic permeability of free space (H m$^{-1}$)
*V*: Volume of magnetic element (m$^{3}$)
$$m_s$$: Saturation magnetization (A m$$^{-1}$$)
*P*: Energy (magnetic or elastic) (J)
$\boldsymbol{\tau }$: Torque (magnetic or elastic) $\in \mathbb {R}^3$ (N m)
$\boldsymbol{u}$: Sleeve constant curvature $\in \mathbb {R}^3$ (rad m$^{-1}$)
*R*: Rotation matrix ∈SO(3) (−)
γ: Axis–angle orientation vector $\in \mathbb {R}^3$ (rad)
α: Lengthwise twist of sleeve 2 (rad)
*K*: Diagonal stiffness matrix $\in \mathbb {R}^{3\times 3}$ (N m$^2$)
*E*: Elastic modulus (N m$$^{-2}$$)
*G*: Shear modulus (N m$$^{-2}$$)
*I*: Area moment of inertia (m$^4$)
*J*: Polar moment of inertia (m$^4$)
*s*: Arc length, measured from the proximal end (m)
*i*: Sleeve index, numbered from outer to inner (−)
*L*: Sleeve total length (m)
β: Planar angle of sleeve tip relative to referential (rad)
